# A Class I Histone Deacetylase Inhibitor Attenuates Insulin Resistance and Inflammation in Palmitate-Treated C2C12 Myotubes and Muscle of HF/HFr Diet Mice

**DOI:** 10.3389/fphar.2020.601448

**Published:** 2020-12-10

**Authors:** Soo Jin Lee, Sung-E Choi, Han Byeol Lee, Min-Woo Song, Young Ha Kim, Jae Yeop Jeong, Yup Kang, Hae Jin Kim, Tae Ho Kim, Ja Young Jeon, Kwan Woo Lee

**Affiliations:** ^1^ Department of Endocrinology and Metabolism, Ajou University School of Medicine, Suwon, South Korea; ^2^ Department of Physiology, Ajou University School of Medicine, Suwon, South Korea; ^3^ Department of Biomedical Science, The Graduate School, Ajou University, Suwon, South Korea; ^4^ Division of Cosmetics and Biotechnology, Hoseo University, Asan-si, South Korea; ^5^ Division of Endocrinology and Metabolism, Department of Internal Medicine, Seoul Medical Center, Seoul, South Korea

**Keywords:** Histone deacetylase inhibitor, MS-275, Insulin resistance, Inflammation, C2C12, Lipotoxicity

## Abstract

Histone deacetylase (HDAC) inhibitors, which regulate gene expression by inhibiting the deacetylation of histones and nonhistone proteins, have been shown to exert a wide array of biological effects; these include anti-cancer, anti-obesity, and anti-diabetes effects, as well as cardiovascular-protective activity. However, the effects of class I HDAC inhibition on lipotoxicity in C2C12 myotubes and skeletal muscle tissue remain poorly understood. In this study, we investigated the molecular mechanism underlying the protective effect of class I HDAC inhibition under lipotoxic conditions, i.e., in palmitate (PA)-treated C2C12 myotubes and skeletal muscle tissue in high fat (HF)/high fructose (HFr) diet mice. PA treatment of C2C12 myotubes increased HDAC3 protein expression and impaired mitochondrial oxidation, resulting in increased mitochondrial ROS generation and an accumulation of intracellular triglycerides (TG). Prolonged exposure led to increased inflammatory cytokine expression and insulin resistance. In contrast, MS-275, a class I HDAC inhibitor, dramatically attenuated lipotoxicity, preventing PA-induced insulin resistance and inflammatory cytokine expression. Similar beneficial effects were also seen following HDAC3 knockdown. In addition, MS-275 increased the mRNA expression of peroxisome proliferator activator receptor γ-coactivator 1α (PGC1α) and mitochondrial transcription factor A (TFAM), which serve as transcriptional coactivators in the context of mitochondrial metabolism and biogenesis, and restored expression of peroxisome proliferator-activated receptor alpha (PPARα), medium-chain acyl-coenzyme A dehydrogenase (MCAD), enoyl-CoA hydratase, and 3-hydroxyacyl CoA dehydrogenase (EHHADH). *In vivo,* treatment of HF/HFr-fed mice with MS-275 ameliorated hyperglycemia, insulin resistance, stress signals, and TNF-α expression in skeletal muscle. Taken together, these results suggest that HDAC3 inhibition rather than HDAC1/2 inhibition by MS-275 protects against lipotoxicity in C2C12 myotubes and skeletal muscle, and may be effective for the treatment of obesity and insulin resistance.

## 1. Introduction

Obesity is a rapidly growing global epidemic, and an important comorbidity of numerous metabolic diseases including diabetes, dyslipidemia, hypertension, and cardiovascular disease (Eckel et al., 2005; Poirier et al., 2006). The link between obesity and insulin resistance is well known. As the primary organ for whole-body glucose absorption and disposal, skeletal muscle accounts for ∼70% of the body’s glucose consumption (Smith and Muscat, 2005). Therefore, any abnormality in glucose metabolism in muscle tissue may induce lipotoxicity, resulting in systemic lipid accumulation and insulin resistance (Petersen et al., 2007; Petersen and Shulman, 2002; DeFronzo and Tripathy, 2009). A tightly regulated balance between fatty acid intake, synthesis, and oxidation is critical for proper lipid metabolism, with prolonged disruption of this balance resulting in lipid accumulation and insulin resistance (Tumova et al., 2016). Ectopic lipid accumulation in muscle tissue produces lipid intermediates such as ceramides, diacylglycerol, and lysophosphatidic acid, which cause oxidative stress, iron dysregulation, and endoplasmic reticulum (ER) stress (Daemen et al., 2018). Fatty acids and various cytokines secreted from intermuscular adipose tissue and peri-muscular adipose tissue have also been shown to activate inflammatory signals (Sachs et al., 2019). This combination of lipid intermediaries and inflammatory signals stimulates serine kinases such as phospho-C-JUN-N-terminal kinase (p-JNK), IkB kinase (IKK), and protein kinase C θ (PKC θ), leading to insulin resistance (Glass and Olefsky, 2012; Wu and Ballantyne, 2017).

Recently, epigenetic dysregulation has been proposed as a key contributor to the development of obesity and diabetes (Ling and Ronn, 2019; Loh et al., 2019), with regulation of these events being a potential treatment target for these conditions (Ling and Ronn, 2019). Previous studies have demonstrated the efficacy of histone deacetylase (HDAC) inhibitors for the treatment of various metabolic diseases, such as obesity, type 1 and type 2 diabetes mellitus (DM), non-alcoholic fatty liver disease (NAFLD), and even chronic kidney disease (CKD) (Zhang et al., 2019; Christensen et al., 2011; Meier and Wagner, 2014). Ferrari *et al.* demonstrated that MS-275, a class 1-specific HDAC inhibitor, dramatically reduced fat mass and adipocyte size in a mouse model of diet-induced obesity (DIO) by increasing the rate of lipolysis and fatty acid *β*-oxidation, resulting in improved glucose tolerance and attenuation of fatty liver disease. Similar effects were seen in db/db mice (Galmozzi et al., 2013). In primary hepatocytes, MS-275 stimulated hepatic expression and secretion of FGF21, which serves as the master regulator of fat oxidation and ketogenesis, via H3K18ac-mediated CREBH signaling (Christensen et al., 2011). MS-275 was also shown to protect human islet cells and MIN6 murine cells against palmitate (PA)-induced cells death via the attenuation of activating transcription factor 3 (Atf3) and C/EBP homologous protein (CHOP) expression (Meier and Wagner, 2014). Finally, MS-275 was shown to attenuate DIO via the induction of white fat browning in mice (Galmozzi et al., 2013). However, the effects of class I HDAC inhibition on free fatty acid-induced insulin resistance and inflammation in the C2C12 myotubes and skeletal muscle of high fat (HF)/high fructose (HFr) diet mice is not known. In this study, we investigated the effects of MS-275 on PA-induced lipotoxicity in C2C12 myotubes and HF/HFr diet mice, and whether these effects were mediated by specific HDAC inhibition.

We found that MS-275 treatment of differentiated C2C12 myotubes improved PA-induced insulin resistance and inflammatory cytokine expression via the inhibition of JNK and nuclear factor-kappa B (NK-κB). Enhanced fatty acid oxidation and mitochondrial function were also observed following attenuation of lipotoxicity by MS-275. Similar beneficial effects were also seen following HDAC3 knockdown. In addition, *in vivo* treatment of HF/HFr-fed mice with MS-275 ameliorated insulin resistance, stress signals, and tumor necrosis factor (TNF)-α expression in skeletal muscle. Together, these results showed that MS-275 improved inflammation and insulin resistance in skeletal muscle, principally by inhibiting HDAC3, and may thus be a promising candidate treatment for obesity and diabetes-related insulin resistance.

## 2. Methods

### 2.1. Cell Culture and Differentiation

Mouse myoblast C2C12 cells were maintained in Dulbecco’s modified Eagle’s medium (DMEM) supplemented with 10% fetal bovine serum (FBS) and antibiotics (10 μg/ml streptomycin and 100 IU/ml penicillin) at 37°C in a 5% CO_2_ atmosphere. C2C12 myoblasts were differentiated in DMEM supplemented with 2% horse serum, with medium changes every 3–5 days. Differentiation states were determined based on morphological changes and the expression of differentiation marker genes.

### 2.2. Reagents

Entinostat (MS-275) was purchased from MedChemExpress (Monmouth Junction, NJ, United States). Other chemicals, including bovine serum albumin (BSA; 2207008), PA (P5585), and insuli n (I9278) were purchased from Sigma-Aldrich (Burlington, MA, United States). 2-(N-(7-nitrobenz-2-oxa-1,3-diazol-4-yl)-amino)-2-deoxyglucose (2*-*NBDG; N13195), 4,4-difluoro-1,3,5,7,8-pentamethyl-4-bora-3a,4a-diaza-s-indacene (BODIPY™ 493/503; D3922), and MitoSOX Red mitochondrial superoxide indicator (M36008) were obtained from Thermo Fisher Scientific (Waltham, MA, United States). Anti-histone deacetylase 1 (HDAC1) (34589), anti-HDAC2 (57156), anti-HDAC3 (85057), anti-phospho-AKT (9271), anti-AKT (9272), anti-phospho-GSK3 α/β (9331), anti-GSK3β (9315), anti-phospho-JNK (9251), anti-JNK (9252), anti-phospho-NF-kB (3033), and anti-NF-kB (3034) antibodies were obtained from Cell Signaling Technology (Beverly, MA, United States). Anti-β-actin (A300-491A) antibody was purchased from Bethyl Laboratories (Montgomery, TX, United States). Anti-α-tubulin (sc-5286) antibody was purchased from Santa Cruz biotechnology (Dallas, TX, United States).

### 2.3. Preparation of PA

Prior to use, 0.01 M NaOH was added to a 20 mM PA solution and incubated at 70°C for 30 min. The resulting fatty acid soap was mixed with 5% BSA in phosphate-buffered saline at a 1:3 volume ratio. BSA/PA conjugates consisting of 3.75% BSA and 5 mM PA were then used to treat differentiated C2C12 cells at the indicated concentrations.

### 2.4. Uptake of 2-NBDG

C2C12 myotubes were pretreated with or without 300 or 400 μM PA for 16 h and starved for 4 h. Cells were then incubated in Krebs-Ringer bicarbonate buffer (pH 7.4) containing 2% BSA at 37°C for 30 min, and then treated with 500 μM 2-NBDG with or without 100 nM insulin at 37°C for 2 h. Collected cells were lyzed with lysis buffer and centrifuged at 12,000 rpm for 30 min. The fluorescence intensity of 2-NBDG in the separated supernatant was measured (excitation: 475 nM; emission: 550 nM) using a SpectraMax iD3 microplate reader (Molecular Devices, Sunnyvale, CA, United States).

### 2.5. Western Blot Analysis

C2C12 myotubes and mouse gastrocnemius muscles were lyzed with RIPA buffer [150 mM NaCl, 1% NP-40, 0.5% deoxycholate, 0.1% sodium dodecyl sulfate (SDS), and 50 mM Tris-Hcl (pH7.5)] supplemented with a protease inhibitor cocktail. Equal concentrations of proteins were diluted in SDS sample buffer (50 mM Tris-Cl at pH 6.8, 2% SDS, 100 mM DL-dithiothreitol (DTT), 10% glycerol), separated on 8–12% polyacrylamide, and transferred to a poly (vinylidene fluoride) (PVDF) membrane sheet. After blocking the membrane in 5% skim milk for 30 min, the target antigen was reacted with the primary antibody at RT for 2 h. The membrane was then incubated with the secondary antibody (horseradish peroxidase-conjugated anti-mouse IgG or anti-rabbit IgG antibodies) at RT for 1 h, after which an immunoreactive band was detected using an enhanced chemiluminescence system (Pierce ECL Western Blotting Substrate; Thermo, Rockford, IL, United States). Band intensity was measured using Quantity One 1D image analysis software (Bio-Rad, Hercules, CA, United States).

### 2.6. Reverse Transcriptase-Polymerase Chain Reaction (RT-PCR)

Total RNA from cells and mouse gastrocnemius muscles was extracted with RNAiso Plus reagent (Takara Bio, Shiga, Japan). cDNA was synthesized using the AMV reverse transcriptase and random 9-mers supplied with the TaKaRa RNA PCR Kit (version 3.0; TaKaRa Bio, Shiga, Japan). The primer sets for PCR amplification are listed in Supplementary Table S1. Quantitative real-time PCR was performed with SYBR Green (TaKaRa Bio) using a TaKaRa TP-815 instrument. Relative quantities of amplified DNA were analyzed using the software bundled with the TP-815 instrument and normalized to mouse 36B4 mRNA levels.

### 2.7. Oxygen Consumption Rate (OCR)

C2C12 myotubes were plated onto XF24 cell culture microplates and differentiated for 3 days in differentiation medium, after which the C2C12 myotubes were either treated or not treated with drugs, depending on the experimental condition. Following treatment, the C2C12 myotubes were pre-washed with Krebs-Ringer bicarbonate (KRB) buffer and equilibrated with XF assay medium supplemented with 2.5 mM glucose/50 mM carnitine/0.2 mM PA (for PA OCR) at 37°C in a CO_2_-free incubator for 1 h. The OCR of PA as a carbon substrate was measured using a XF24 extracellular analyzer (Seahorse Bioscience, North Billerica, MA, United States).

### 2.8. Transfection of Small Interfering RNA (siRNA)

Small interfering RNA duplexes were designed and synthesized by Bioneer Corporation (Daejeon, Korea). The sequences were as follows: green fluorescent protein (GFP) (GenBank: GU983383), sense-GUU CAG CGU GUC CGG CGA, antisense- CUC GGC GGA CAC GCU GAA C; mouse HDAC1 (GenBank: NM_008228.2), sense-GAG GUU GAU AGC CUA GCU U, antisense-AAG CUA GGC UAU CAA CCU C; mouse HDAC2 (GenBank:NM_008229.2), sense-UCA GAC AAA CGG AUA GCU U, antisense-AAG CUA UCC GUU UGU CUG A; mouse histone deacetylase 3 (HDAC3) (GenBank:NM_010411.2), sense-CAC AGA GAC UGU UAG AGA U, antisense-AUC UCU AAC AGU CUC UGU G. C2C12 cells were transfected with siRNA duplex using a Neon^™^ electro-transfection system (Invitrogen, Carlsbad, CA, United States). siRNA duplexes were suspended in R buffer supplied in the Neon™ electro-transfection system and transfected into C2C12 myoblasts under a pulse voltage of 1,005 V, with a pulse width of 35 ms and pulse number of 2. Transfected C2C12 myoblasts were then seeded into growth plates and incubated for 16 h, and then differentiated in differentiation medium for 3 days followed by treatment with 300 or 400 μM PA (or no treatment) for 12 h.

### 2.9. Animal Studies

All animal experiments were approved by the Animal Ethics Committee of Ajou University (Permission number: 2018–0030). Six-week-old male C57BL/6J mice were purchased from GEM Pharmatechnology (Nanjing, China). The mice were housed in a temperature-controlled room at 22 ± 2°C with a light/dark cycle of 12 h and fed *ad libitum*. After 2 weeks of adaptation, 8-week-old mice were randomly divided into three groups: 1) DMSO-injected control diet group (CD/DMSO) (*n* = 5); 2) DMSO-injected high fat (HF)/high fructose (HFr) diet group (HF/HFr/DMSO) (*n* = 5); and 3) MS-275-injected HF/HFr diet group (HF/HFr/MS-275) (*n* = 5). The CD mice were fed normal chow diet containing 10% fat (D12450B; Research Diets Inc., New Brunswick, NJ, United States) and water. HF/HFr mice were fed a diet containing 60% fat (D12492; Research Diets Inc.) and drinking water including 30% fructose. Mice were injected intraperitoneally every other day with DMSO or 10 mg/kg MS-275 for 11 weeks. Insulin tolerance test (ITT) and glucose tolerance test (GTT) were performed during week 10 of the diet. Mice were fasted for 6 h and insulin (0.7 U/kg) or glucose (1 g/kg) was injected intraperitoneally. Blood was collected from the tail at the indicated time points (0, 15, 30, 60, and 90 min), with glucose levels measured using an Accu-Chek device (Roche Diagnostics, Mannheim, Germany).

### 2.10. Intracellular Triglyceride Staining

Intracellular triglyceride (TG) levels were determined by measuring relative fluorescence intensity in cells after treatment with BODIPY. Briefly, C2C12 myotubes were pretreated with MS-275 for 16 h in a dose-dependent manner. Cells were then treated with 500 uM PA for 3 h, washed with PBS buffer (137 mM NaCl, 2.7 mM KCl, 10 mM Na_2_HPO_4_, 1.8 mM KH_2_PO_4_), and incubated in 100 μL of 2.0 μM BODIPY at 37°C for 30 min. Fluorescence intensity was measured at 430 and 510 nm (exciting and emitting wavelengths, respectively) using a SpectraMax iD3 microplate reader (Molecular Devices).

### 2.11. Mitochondrial Superoxide Staining

After treatment with MitoSOX Red, mitochondrial superoxide levels were determined by measuring relative the fluorescence intensity in C2C12 myotubes. Mitochondrial superoxide in C2C12 myotubes was measured using the same protocol as for intracellular TG. Fluorescence intensity was measured by fluorescence spectrophotometry at 510 and 580 nm (exciting and emitting wavelengths, respectively).

### 2.12. Insulin Measurement

Plasma insulin levels were measured using the Shibayagi Mouse Insulin ELISA kit (Cunma, Japan). Briefly, blood obtained from the mouse tail vein was immediately centrifuged at 3,000 × *g* for 10 min at 37°C. The supernatant plasma was collected and stored at -80°C. A biotinylated-anti-insulin antibody solution (100 μL) was added to each well of a 96-well plate coated with an anti-insulin antibody and mixed with 10 μL of plasma. After 2 h incubation at room temperature, the solution containing the insulin/biotinylated-anti-insulin antibody complex was removed. Biotinylated-anti-insulin antibody bound to insulin coated onto the plate was incubated with 100 μL of horse radish peroxidase (HRP)-streptavidin solution (one of the kit components) for 30 min at room temperature. After removal of unbound HRP-streptavidin, the HRP-streptavidin bound to the plate was reacted with 3, 3’, 5, 5’–tetramethylbenzidine (TMB) in 100 μL of the chromogen solution. After the reaction was stopped with 100 μL 1 M sulfuric acid, absorbance at 450 nm was measured using a microplate reader (Bio-Rad, Hercules, CA, United States). The plasma insulin levels were calculated using an insulin standard curve. The homeostasis model for insulin resistance (HOMA-IR) was calculated as the fasting blood glucose level (mg/dl) × the fasting plasma insulin level (µU/ml) divided by 405.

### 2.13. Statistical Analysis

All experiments were repeated at least three times. All data are expressed as the mean ± SE and were analyzed using GraphPad Prism 6.0 (GraphPad Software Inc., San Diego, CA, United States). One-way analysis of variance (ANOVA) with the Bonferroni post hoc test was used. *p* values < 0.05 were considered statistically significant.

## 3. Results

### 3.1. PA Induces Mitochondrial Dysfunction and Lipotoxicity in C2C12 Myotubes

**FIGURE 1:**
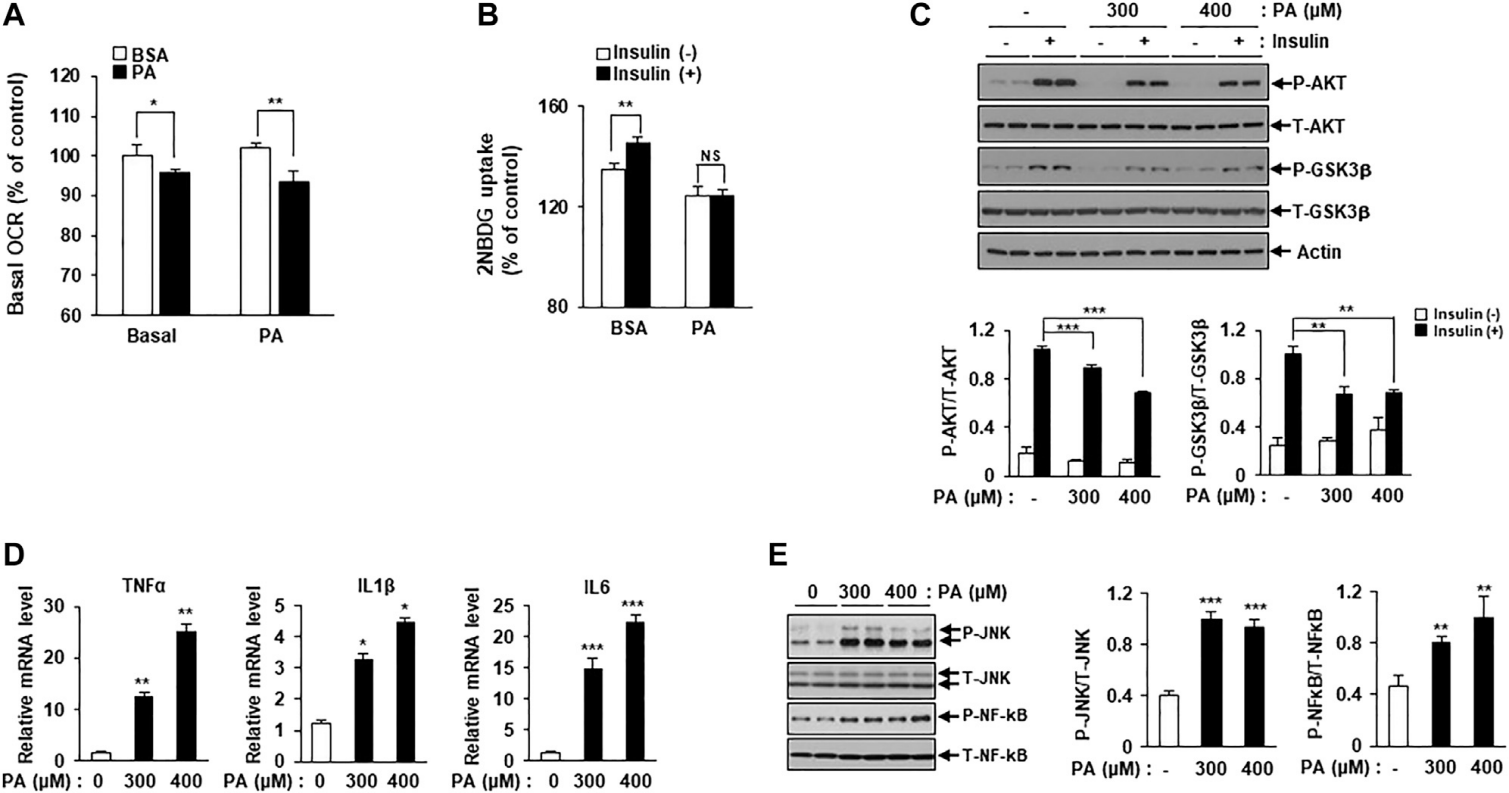
PA induces mitochondrial dysfunction and lipotoxicity. **(A)** Differentiated C2C12 myotubes were treated with or without 400 μM PA for 12 h. The OCR of PA was measured using the Seahorse XF Analyzer (Agilent). *p < 0.05; **p < 0.01 vs. PA-untreated cells. **(B)** C2C12 myotubes were treated with 400 μM PA for 12 h, and then stimulated with insulin at 100 nM for 3 h. 2-NBDG uptake was measured using a SpectraMax iD3 microplate reader (excitation: 475 nM; emission: 550 nM). **p < 0.01 vs. 2-NBDG uptake after insulin treatment. **(C)** After treatment with 300 or 400 μM PA, insulin resistance in C2C12 myotubes was determined by measuring p-AKT and p-GSK3β by Western blot. **p < 0.01; ***p < 0.001 vs. insulin-stimulated and PA-untreated cells. **(D)** C2C12 myotubes were incubated with 300 or 400 μM PA for 12 h, after which inflammatory cytokine gene expression was measured by real-time PCR. *p < 0.05; ***p < 0.001 vs. PA-untreated cells. **(E)** The expression levels of the inflammatory protein markers p-JNK and p-NF-kB were measured by Western blot. **p < 0.01; ***p < 0.001 vs. PA-untreated cells.

An increasing body of evidence suggests an association between mitochondrial dysfunction and lipotoxicity, which is implicated in insulin resistance and inflammation. To detect mitochondrial failure in PA-treated C2C12 myotubes, we measured PA oxidation using the Seahorse Extracellular Flux (XF) Analyzer (Agilent, Santa Clara, CA, United States). Mitochondrial OCR was reduced in PA-treated C2C12 myotubes compared to BSA-treated myotubes (Figure 1A).

Next, we confirmed lipotoxicity induction in C2C12 myotubes following treatment with PA. The addition of 300 or 400 μM PA for 12 h was shown to induce insulin resistance and impair insulin-stimulated glucose uptake. 2-NBDG uptake increased in response to insulin stimulation compared to the unstimulated myotubes, while 2-NBDG uptake in PA-treated myotubes remained unchanged (Figure 1B). Levels of various insulin signaling molecules, including phospho-protein kinase B (p-AKT) and phospho-glycogen synthase kinase three *ß* (p-GSK3β) were reduced by PA treatment in a dose-dependent manner (Figure 1C). The expression levels of inflammatory cytokines, such as TNF-α, interleukin (IL)-1β, and IL-6 were increased by PA treatment (Figure 1D). Furthermore, PA increased the expression of stress and inflammatory markers, such as p-JNK and p-NF-κB, in C2C12 myotubes (Figure 1E). Together, these results suggested that PA induced mitochondrial dysfunction and lipotoxicity in C2C12 myotubes, leading to significant increases in insulin resistance and inflammation.

### 3.2. MS-275 Attenuated PA-Induced Insulin Resistance and Inflammation

**FIGURE 2:**
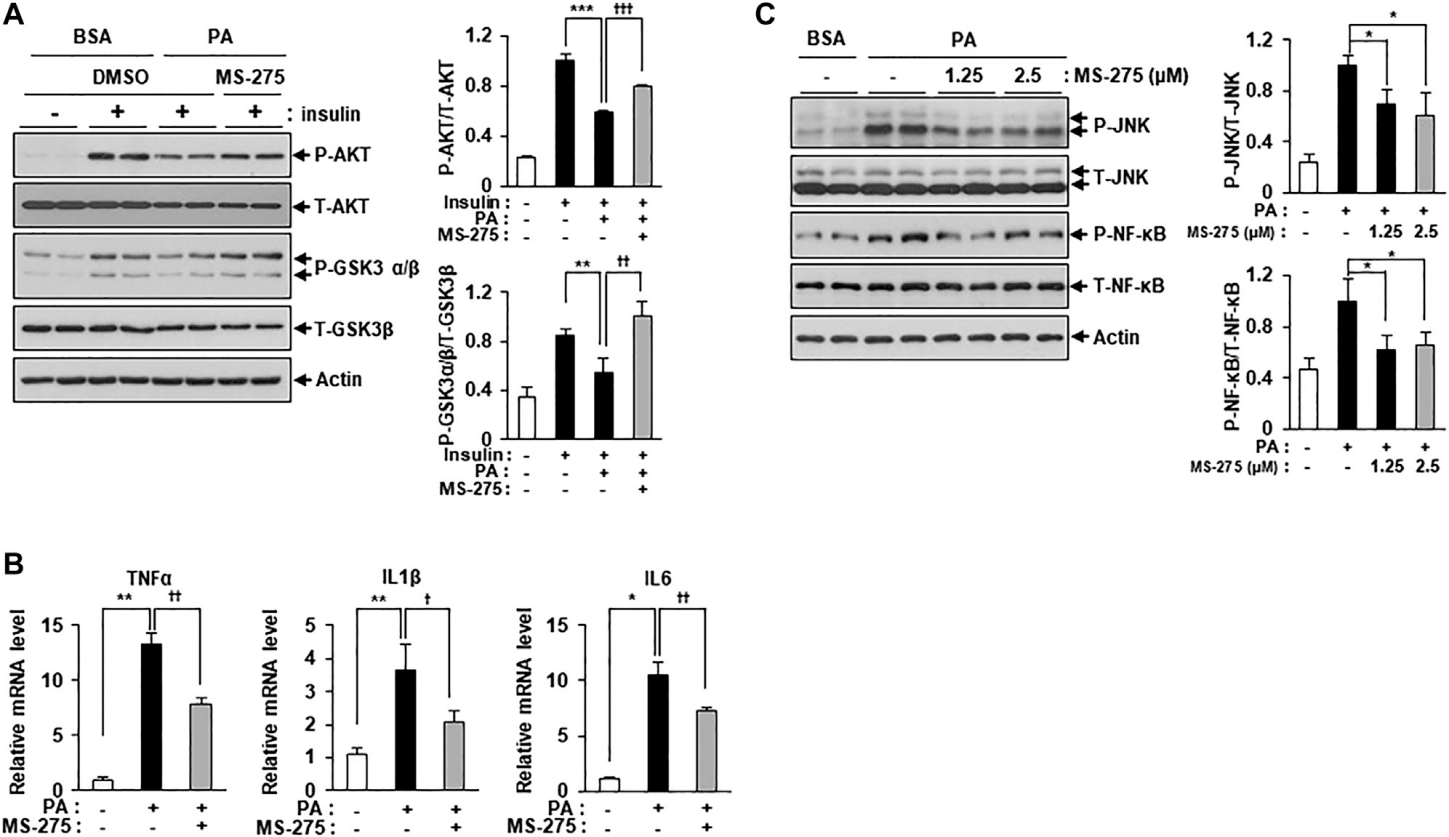
MS-275 restored PA-induced insulin resistance and inflammation in C2C12 myotubes. **(A)** C2C12 myotubes were treated with 2.5 μM MS-275 in the presence of 300 μM PA for 12 h, and then stimulated with 100 nM insulin for 20 min. Cells were then harvested and analyzed by Western blot. The levels of insulin signaling-related proteins (p-AKT and p-GSK3β) were detected by Western blot; **p < 0.01; ***p < 0.001 vs. insulin-stimulated and PA-untreated cells, ^††^ *p* < 0.01; †††*p* < 0.001 vs. insulin-stimulated and PA-treated cells. **(B)** After treatment with 300 μM PA with or without 2.5 μM MS-275 for 12 h, the expression of inflammatory genes (TNF-α, IL-1β, and IL-6) was quantified by real-time PCR. *p < 0.05; **p < 0.01 vs. PA-untreated cells, †*p* < 0.05; ^††^ *p* < 0.01 vs. PA-treated cells. **(C)** C2C12 myotubes were treated with the indicated dosage of MS-275 in the presence of 300 μM PA for 12 h. The levels of stress/inflammation-related proteins were analyzed by Western blot using antibodies against p-JNK and p-NF-κB. *p < 0.05 vs. PA-treated cells.

To explore whether inhibition of class I HDACs via treatment with MS-275 could protect against lipotoxicity, such as that seen in PA-treated C2C12 myotubes, we added PA with or without MS-275. MS-275 treatment significantly restored the expression of insulin signaling markers, including p-AKT and p-GSK3β, which were reduced by PA (Figure 2A). The expression levels of inflammatory cytokine genes, such as TNF-α, IL-1β, and IL-6, were also increased by PA treatment but this was attenuated by MS-275 treatment (Figure 2B). MS-275 treatment of C2C12 myotubes was also shown to reduce levels of inflammatory markers, such as p-JNK and p-NF-κB, which were increased by PA (Figure 2C). Together, these data demonstrated that MS-275 had a protective effect against lipotoxicity, preventing PA-induced insulin resistance and inflammation in C2C12 myotubes.

### 3.3. HDAC3 Protein Levels, but Not Those of HDAC1 or HDAC2, Were Significantly Increased in PA-Treated C2C12 Myotubes

**FIGURE 3:**
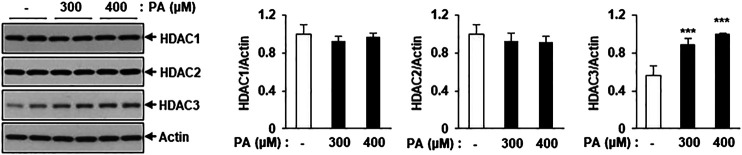
Treatment of PA increased the class I HDACs protein level in C2C12 myotubes. C2C12 myotubes were incubated with 300 or 400 μM PA for 12 h and class1 HDAC protein levels were detected by Western blot. ***p < 0.001 vs. PA-untreated cells.

To detect changes in the levels of class I HDAC family members, C2C12 myotubes were treated with PA, followed by assessment of HDAC1, HDAC2, and HDAC3 protein levels via immunoblotting. HDAC3 protein expression was significantly increased in PA-treated myotubes compared to untreated myotubes, but HDAC1 and HDAC2 protein levels did not increase (Figure 3). The increase in HDAC3 protein expression may play a role in the PA-induced lipotoxicity evident in C2C12 myotubes.

### 3.4. Inhibition of HDAC3 Prevented PA-Induced Insulin Resistance and Inflammation

**FIGURE 4:**
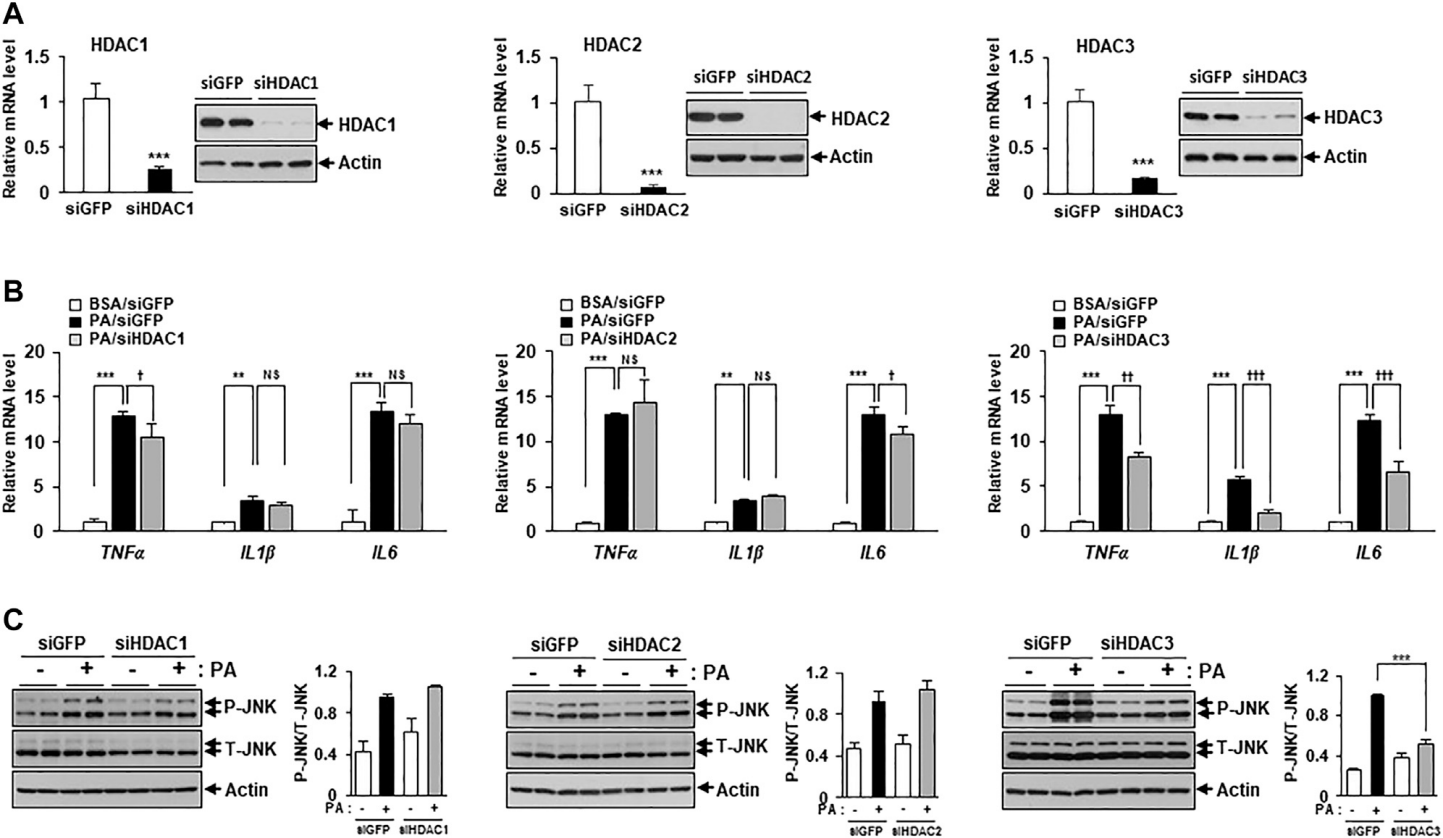
HDAC3 knockdown improved PA-induced inflammation. **(A)** C2C12 myotubes were transfected with HDAC1, HDAC2, HDAC3, or GFP (control) siRNAs. After incubation in differentiation media for 48 h, knockdown of HDAC1, HDAC2 or HDAC3 was determined by real-time PCR and Western blot. ***p < 0.001 vs. GFP-transfected cells. Transfected C2C12 myotubes were treated with 300 μM PA for 12 h. The induction of inflammatory genes (TNF-α, IL-1β, and IL-6) was determined by real-time PCR **(B)** and stress/inflammatory signal activation was determined by measuring the levels of p-JNK by Western blot **(C)**. ***p < 0.001 vs. GFP-transfected cells, ^††^ *p* < 0.01; ^†††^ *p* < 0.001 vs. GFP-transfected and PA-treated cells.

Figure 2 showed that MS-275 exhibited strong protective effects against PA-induced lipotoxicity, including insulin resistance and inflammation. Since MS-275 inhibits HDAC1, HDAC2, and HDAC3, we aimed to determine which HDAC was involved in this protective effect. To identify the main effector protein, C2C12 myotubes were transfected with HDAC1, HDAC2, or HDAC3 small interfering RNAs (siRNAs), and cytokine expression and inflammatory signaling were assessed. Class I HDAC knockdown was confirmed by real-time PCR and immunoblotting. The expression levels of HDAC1, HDAC2, and HDAC3 were significantly reduced (Figure 4A). The PA-induced increases in the expression levels of TNF-α, IL-1β, and IL-6 were significantly reduced on transfection of HDAC3 siRNA. HDAC1 knockdown slightly reduced only the TNF-α level, and HDAC2 knockdown only that of IL6 (Figure 4B). Similarly, the level of the inflammatory marker p-JNK was attenuated by HDAC3 siRNA only (Figure 4C). Next, we investigated the role of insulin signaling, with or without insulin treatment, via Western blot. After insulin stimulation, the levels of p-AKT and p-GSK3β were significantly increased in HDAC3 knockdown myotubes compared to siGFP-transfected C2C12 myotubes. Moreover, this reduction in insulin signaling following PA treatment was restored in HDAC3 knockdown myotubes (Supplementary Figure S1). Together, the data suggest that HDAC3 inhibition of MS-275 protected against PA-induced lipotoxicity.

### 3.5. MS-275 Reduced Intracellular TG via Induction of Mitochondrial Fatty Acid Oxidation

**FIGURE 5:**
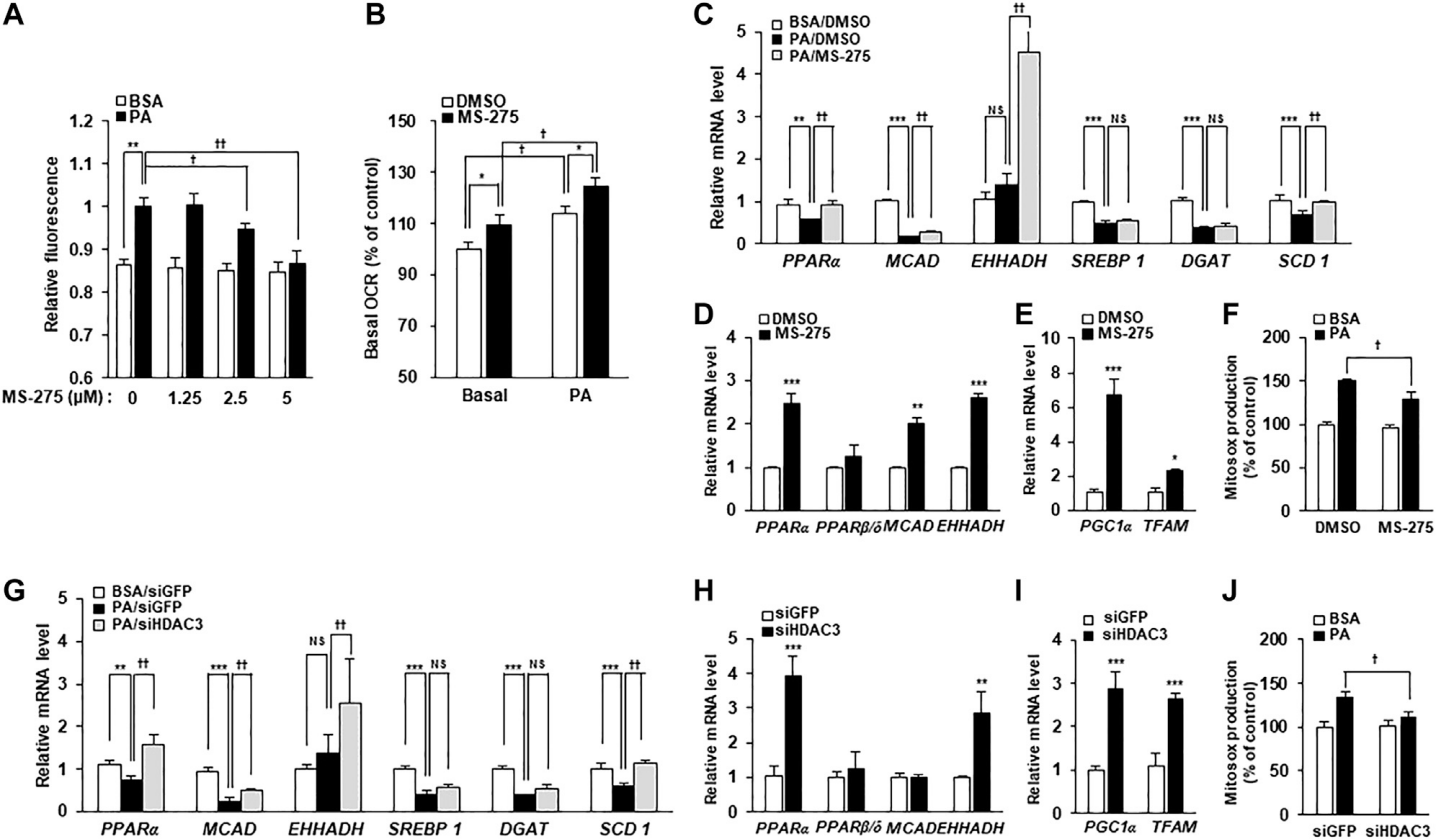
Inhibition of HDAC3 by MS-275 or siRNA increased mitochondrial fatty acid oxidation and related gene expression. **(A)** C2C12 myotubes were pretreated with various concentrations of MS-275 for 16 h and then treated with 300 μM PA for 6 h. The intracellular TG content was determined by measuring the fluorescence of BODIPY. **p < 0.01 vs. PA-untreated cells, ^†^ *p* < 0.05; ^††^ *p* < 0.01 vs. PA-treated cells. **(B)** The OCR of PA when used as the sole carbon substrate was measured using the Seahorse XF Analyzer (Agilent). *p < 0.05 vs. MS-275-untreated cells, †*p* < 0.05 vs. PA-untreated cells. **(C)** C2C12 myotubes were treated with or without 2.5 μM MS-275 in the presence of 300 μM PA for 12 h. The expression of fatty acid metabolism-related genes was determined by real-time PCR. ***p < 0.001 vs. PA-untreated cells, ^††^ *p* < 0.01 vs. PA-treated cells. **(D)** After treatment of 2.5 µM MS-275 for 16 h, the expression of fatty acid oxidation-related genes was measured by real-time PCR. **p < 0.01; ***p < 0.00 vs. MS-275-untreated cells. **(E)** C2C12 myotubes were treated with 2.5 µM MS-275 for 16 h, and mitochondrial metabolism/biogenesis related gene expression was then measured using real-time PCR. *p < 0.05; **p < 0.01; ***p < 0.00 vs. MS-275-untreated cells. **(F)** C2C12 myotubes were pretreated with 2.5 µM MS-275 for 16 h and then treated with 300 μM PA for 4 h. Mitochondrial superoxide was determined by measuring the fluorescence of MitoSOX Red. **p < 0.01 vs. PA-untreated cells, ^†^ *p* < 0.05; ^††^ *p* < 0.01 vs. PA-treated cells. **(G)** C2C12 myotubes were transfected with HDAC3 siRNA and then 300 μM PA was treated for 12 h. The expression of fatty acid metabolism-related genes was determined by real-time PCR. ***p < 0.001 vs. PA-untreated cells, ^††^ *p* < 0.01 vs. PA-treated cells. **(H and I)** After transfection with HDAC3 siRNA, C2C12 myotubes were differentiated for 3 days, and then the expression of fatty acid oxidation-related genes or mitochondrial metabolism/biogenesis related genes, respectively were measured by real-time PCR. **p < 0.01; ***p < 0.00 vs. GFP-transfected cells. **(J)** After transfection with HDAC3 siRNA, C2C12 myotubes were differentiated for 3 days, and then treated with 300 μM PA for 4 h. Mitochondrial superoxide was determined by measuring the fluorescence of MitoSOX Red. **p < 0.01 vs. PA-untreated cells and GFP-transfected cells, ^†^ *p* < 0.05; ^††^ *p* < 0.01 vs. PA-treated cells and GFP-transfected cells.

To investigate the mechanisms underlying the protective effect of MS-275 on PA-induced insulin resistance and inflammation, PA-induced fat accumulation was investigated via staining of intracellular TGs using BODIPY dyes. Treatment with MS-275 was shown to reduce PA-induced TG accumulation in a dose-dependent manner (Figure 5A). After adding PA as a carbon substrate in C2C12 myotubes, fatty acid oxidation was measured as a function of OCR using the XF Analyzer. Pretreatment of MS-275 for 16 h increased OCR in C2C12 myotubes compared to untreated controls (Figure 5B). Next, we investigated gene expression in relation to fatty acid oxidation and lipid synthesis. Treatment of PA decreased the expression levels of fatty acid oxidation-related genes, such as proliferator-activated receptor alpha (PPARα) and medium-chain acyl-coenzyme A dehydrogenase (MCAD). In contrast, MS-275 treatment significantly rescued the expression of fatty acid oxidation-related genes and stearoyl-CoA desaturase 1 (SCD1). Sterol regulatory element-binding protein 1 (SREBP1) and diglyceride acyltransferase (DGAT) were significantly reduced following PA treatment, but were unchanged by MS-275 (Figure 5C). Furthermore, MS-275 treatment in C2C12 without PA was found to increase the expression of PPARα, MCAD, and EHHADH (Figure 5D). In addition, MS-275 increased the expression of PGC1α and mitochondrial transcription factor A (TFAM) genes. PGC1α and TFAM are used as markers of transcriptional coactivation in the context of mitochondrial metabolism and biogenesis (Figure 5E). In addition, MS-275 decreased PA-stimulated MitoSOX Red staining, which is used as a marker of mitochondrial reactive oxygen species (ROS) generation (Figure 5F). To investigate the mechanisms underlying the protective effect of HDAC3 knockdown on PA-induced insulin resistance and inflammation, we performed an experiment similar to that described above. PA treatment decreased PPARα, MCAD, SREBP1, DGAT, and SCD1 levels. As was also true of MS-275, HDAC3 knockdown significantly rescued the PA-induced decreases in PPARα, MCAD, EHHADH, and SCD1 gene expression (Figure 5G). In addition, HDAC3 knockdown in C2C12 cells (in the absence of PA) increased the expression of PPARα, EHHADH, PGC1α, and TFAM (Figures 5H,I). Furthermore, PA-stimulated mitochondrial ROS generation reduced on transfection of HDAC3 siRNA compared to transfection of green fluorescent protein (GFP) siRNA (Figure 5J). Song et al. published similar results (Song et al., 2019). Together, the data suggest that MS-275 treatment and HDAC3 knockdown increased the expression of fat oxidation-related genes, reduced mitochondrial ROS production, and restored mitochondrial function. Thus, MS-275 and HDAC3 knockdown promoted fatty acid oxidation by improving mitochondrial metabolism and biogenesis.

### 3.6. MS-275 ameliorated HF/HFr-Induced Insulin Resistance and Inflammation in Skeletal Muscle

**FIGURE 6:**
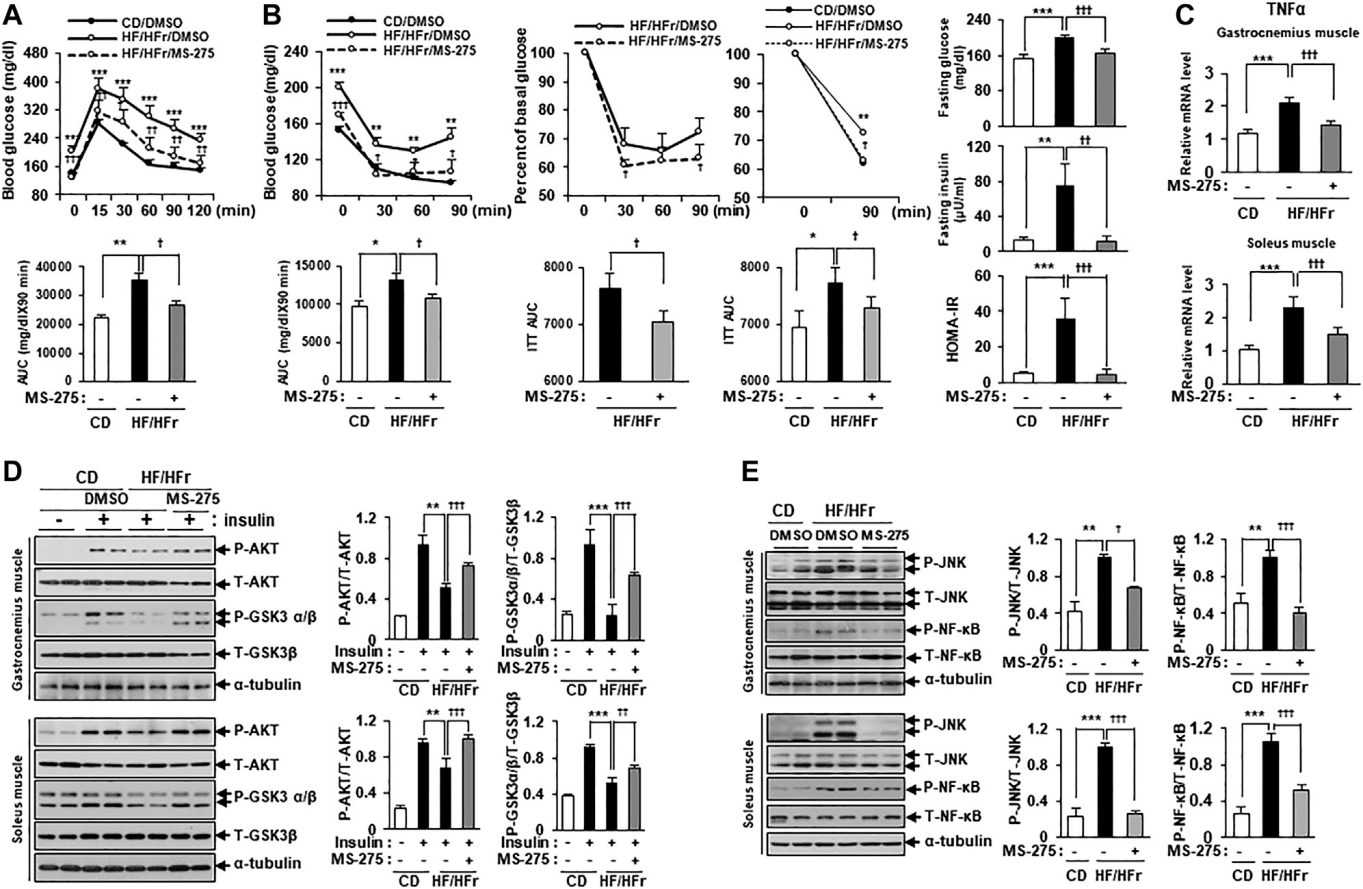
MS-275 ameliorated HF/HFr-induced insulin resistance and inflammation in skeletal muscle from C57BL/6J mice. **(A)** To assess blood glucose levels, mice were fasted for 6 h and then intraperitoneally injected with glucose (1 g/kg). Blood glucose levels were measured at the indicated times using an Accu-Chek glucometer (Roche Diagnostics). The area under the curve (AUC) was obtained from an intraperitoneal glucose tolerance test (IPGTT). **p < 0.01; ***p < 0.001 vs. CD mice, ^†^ *p* < 0.05; ^††^ *p* < 0.005; ^†††^ *p* < 0.001 vs. HF/HFr mice. **(B)** Mice were fasted for 6 h and then intraperitoneally injected with insulin (0.7 U/kg) and then Blood glucose level were analyzed by ACCU-CHEK during IPITT. AUC was obtained from an intraperitoneal insulin tolerance test (IPITT). *p < 0.05; **p < 0.01; ***p < 0.001 vs. CD mice, ^†^ *p* < 0.05; ^†††^ *p* < 0.001 vs. HF/HFr mice. Percent of basal glucose level and the inverse integrated area under the glucose curve during IPITT. *p < 0.05; **p < 0.01 vs. CD mice, ^†^ *p* < 0.05 vs. HF/HFr mice. To assess fasting glucose and insulin levels, after serum sample collection, plasma insulin concentration was analyzed by ELISA described in Methods. HOMA-IR was calculated as described in Methods. **p < 0.01; ***p < 0.001 vs. CD mice, ^††^ *p* < 0.01; ^†††^ *p* < 0.001 vs. HF/HFr mice. **(C)** The relative expression of TNFα gene related to inflammation in gastrocnemius muscle or soleus muscle, respectively was determined by real-time PCR. ***p < 0.001 vs. CD mice, ^†††^ *p* < 0.001 vs. HF/HFr mice. **(D)** Levels of insulin signaling molecules after insulin stimulation in gastrocnemius muscle or soleus muscle, respectively were analyzed by Western blot using anti-p-AKT and anti-p-GSK3β antibodies. **p < 0.01; ***p < 0.001 vs. insulin-stimulated CD mice, ^††^ *p* < 0.01; ^†††^ *p* < 0.001 vs. insulin-stimulated HF/HFr mice. **(E)** The levels of stress/inflammation signaling molecules in gastrocnemius muscle or soleus muscle, respectively were analyzed by Western blot using anti-p-JNK and anti-p-NF-κB antibodies. **p < 0.01; ***p < 0.001 vs. CD mice, ^†^ *p* < 0.05; ^†††^ *p* < 0.001 vs. HF/HFr mice.

Increased HF intake and HFr consumption have previously been shown to contribute to insulin resistance in mice (Han et al., 2016). As MS-275 was found to confer a strong protective effect against PA-induced lipotoxicity in C2C12 myotubes, we further investigated the protective effect of MS-275 in the muscle of HF/HFr diet mice. C57BL/6J mice were randomly divided into three groups: a standard CD group, an HF/HFr diet group, and an HF/HFr group treated with MS-275 (HF/HFr/MS-275). All HF/HFr mice were fed a diet consisting of 60% fat along with 30% fructose water (HF/HFr) for 11 weeks. Each group was intraperitoneally administered either dimethyl sulfoxide (DMSO) or MS-275 (10 mg/kg) every other day for 11 weeks (Supplementary Figure S2). As shown in Figure 6A, the intraperitoneal glucose tolerance test (IPGTT) was performed during week 10 of the dietary regime. The blood glucose level at this time, and the area under the curve (AUC) value of mice on the HF/HFr diet, were increased compared to those of mice on the control diet. However, MS-275-treated mice exhibited significant reductions in these parameters (Figure 6A). The intraperitoneal insulin tolerance test (IPITT) was also performed during week 10 of the dietary regime. The blood glucose level at this, time and the AUC in MS-275-treated mice, were significantly lower compared to those of mice on the HF/HFr diet. The basal glucose levels at this time are shown in Figure 6B right panel. The fasting glucose and insulin levels increased in HF/HFr-diet mice, as did the HOMA-IR. However, MS-275-treated mice exhibited significantly lower fasting glucose and insulin levels, and a lower HOMA-IR, compared to HF/HFr-diet mice (Figure 6B). The TNF-α expression level was reduced in the MS-275-treated gastrocnemius and soleus muscles of HF/HFr-diet mice (Figure 6C). The phospho-AKT and phospho-GSK-3α/β levels in mice on the HF/HFr diet decreased compared to those of insulin-stimulated CD mice. However, the p-AKT and p-GSK-3α/β levels of the gastrocnemius and soleus muscles of HF/HFr/MS-275-diet mice were increased compared to those of insulin-stimulated mice on the HF/HFr diet (Figure 6D). In addition, levels of markers such as p-JNK and p-NF-κB were decreased by MS-275 treatment (Figure 6E). These data suggested that treatment of HF/HFr-diet mice with MS-275 significantly attenuated hyperglycemia, insulin resistance, and inflammation in skeletal muscle both *in vivo* and *in vitro*.

## 4. Discussion

Obesity and systemic lipid overflow are strongly associated with insulin resistance (Samuel and Shulman, 2016). Skeletal muscle, which is the main target site of insulin, plays an important role in the development of insulin resistance (Samuel and Shulman, 2016). Impaired fatty acid oxidation and increased fatty acid content in skeletal muscle results in an accumulation of intracellular fat in skeletal muscle and chronic low-grade inflammation (van der Kolk et al., 2016; Meex et al., 2019). This accumulation of excess fat in skeletal muscle leads to mitochondrial dysfunction and inflammation, which is associated with insulin resistance (Hoeks and Schrauwen, 2012; Meex et al., 2019). Previous studies showed that TSA and sodium butyrate, which function as pan-HDAC inhibitors, improved insulin signaling in mouse skeletal muscle (Sun and Zhou, 2008; Chriett et al., 2019). Here, we assess the effects of MS-275, a class I-specific HDAC inhibitor, on insulin resistance and inflammatory signaling in skeletal muscle. The data presented here provide strong evidence that MS-275 prevented PA-induced insulin resistance and reduced the expression of inflammatory cytokines in differentiated C2C12, by increasing mitochondrial oxidation and lipid oxidation-related gene expression. Over time, MS-275 was shown to reduce PA-induced accumulation of cellular TGs, attenuated mitochondrial ROS, and restored mitochondrial biogenesis. PA treatment of C2C12 myotubes significantly increased HDAC3 protein levels, but not those of HDAC1 or HDAC2. Knockdown of HDAC3 exhibited similar beneficial effects. In addition, MS-275 treatment of HF/HFr-fed mice ameliorated insulin resistance, stress-related signaling, and TNF-α expression in skeletal muscle. The data presented here suggest that treatment with MS-275 may help restore lipotoxicity-induced insulin resistance and inflammation in skeletal muscle via the inhibition of HDAC3, rather than HDAC1/2.

While the beneficial metabolic effects of MS-275 have been demonstrated in several animal models (Galmozzi et al., 2013; Ferrari et al., 2017), the effects of MS-275 appear to differ depending on the animal model and experimental design. Galmozzi *et al.* suggested that HDAC inhibitors enhanced energy metabolism in skeletal muscle and adipose tissue, resulting in reduced body weight in db/db mice. Ferrari reported MS-275 improved glucose tolerance by acting on adipose tissue, but not liver or muscle. They found no improvement in insulin-tolerance in DIO mice. Here, we injected MS-275 intraperitoneally in HF/HFr diet mice every other day for 11 weeks, and found that treatment with MS-275 reduced body weight and improved both glucose tolerance and insulin sensitivity. Moreover, MS-275 restored the levels of major insulin signals such as p-AKT and p-GSK. Possible explanations for these differences include differences in diet (HF vs. HF/HFr diet), and in the initiation, timing, and duration of MS-275 treatment (every other day for 22 days after induction of obesity in Ferrari study vs. every other day for 11 weeks before induction of obesity in this study). Based on this experiment, MS-275 may be expected to have a better metabolic and preventive effect when administered to high-risk groups before obesity induction.

Histological experiments showed that fatty liver was dramatically improved in MS-275-treated mice compared to HF/HFr-diet mice (data not shown). Although we focused on the effects of MS-275 on skeletal muscle, improvement of glucose tolerance and insulin tolerance by MS-275 *in vivo* suggests a diverse activity profile, with improvements in disease-related conditions in skeletal muscle as well as liver.

A recent study reported transcriptional effects of butyrate, a pan-HDAC inhibitor, on PA-induced insulin resistance in L6 rat muscle cells (Chriett et al., 2019). In this study, we also used PA to induce insulin resistance and found that MS-275 was able to protect against lipotoxicity in C2C12 myotubes. Increased concentrations of free fatty acids in plasma suggests a metabolic link between obesity and insulin resistance (Boden et al., 1994). PA, as a saturated fatty acid, is one of the most abundant fatty acids in the blood and is produced via result of lipolysis or lipogenesis (Nolan and Prentki, 2008). PA treatment is thought to mimic the lipotoxicity associated with obesity. In our study, PA induced the expression of various markers of stress and inflammation. Treatment with MS-275 reduced mitochondrial reactive oxygen species (ROS) generation and the activation of JNK and NK-κB by PA in C2C12 myotubes. These results suggest that MS-275 prevented PA-induced insulin resistance via regulation of the mitochondrial ROS/JNK axis. The effect of MS-275 on PA-induced lipotoxicity is consistent with that seen in beta cells, and is mediated by reduced Atf3 and CHOP expression (Plaisance et al., 2014).

We found that MS-275 exerted anti-inflammatory effects on skeletal muscles, associated with suppression of NF-κB and JNK activity. The effects of HDAC inhibitors, including MS-275, on the NF-κB and MAP signaling pathways are complex and may vary among cell types (Leus et al., 2017; Dai et al., 2005). HDAC inhibitors affected these signaling pathways in immune cells such as macrophages, but not in synovial or gingival fibroblasts (Lagosz et al., 2019; Grabiec et al., 2011; Dai et al., 2005). Previous studies reported the effects of HDAC inhibitors on NF-κB or MAP signaling in the context of insulin resistance and skeletal muscle atrophy (Chriett et al., 2017; To et al., 2017). HDAC inhibitors, such as MS-275, are thought to affect NF-kb and JNK signaling in skeletal muscle as in macrophages.

MS-275, as a class 1-specific HDAC inhibitor, has been shown to primarily inhibit HDACs 1, 2 and 3 (Ye, 2013). In our study, the effect of MS-275 on PA-induced lipotoxicity in C2C12 myotubes appears to be mediated by inhibition of HDAC3 rather than HDAC1 or HDAC2, as evidenced by the enhancement of only the HDAC3 protein level by PA, the restoration of insulin-signaling, reduction in inflammatory cytokine production, and recovery of the expression of fatty acid oxidation-related genes in HDAC3 knockdown myotubes. These results are broadly consistent with previous studies (Galmozzi et al., 2013). One study reported that a 70% reduction in HDAC3 expression is sufficient to mimic the effect of class I HDAC inhibitors on PGC1α, glucose transporter 4, TFAM, and isocitrate dehydrogenase 3α (IDH3α) expression, while silencing of HDAC1 had no effect on the expression of these genes in C2C12 (Galmozzi et al., 2013). MS-275 treatment was also shown to enhance mitochondrial activation and oxidative metabolism in skeletal muscle and adipose tissue, and improved obesity and diabetes in db/db mice (Galmozzi et al., 2013). Depletion of HDAC3 in knockout mice models restored normal metabolic activity, as reflected in improved systemic insulin sensitivity in Hdac3-/Osx1+, enhancement of oxidative metabolism in skeletal muscle-specific HDAC3-depleted mice, and prevention of DIO in intestinal epithelial cells (Hong et al., 2017; McGee-Lawrence et al., 2018; Whitt et al., 2018). In addition, patients with type 2 diabetes showed higher HDAC3 expression in peripheral blood mononuclear cells, along with simultaneous activation of pro-inflammatory markers and insulin resistance (Sathishkumar et al., 2016).

In conclusion, our study showed that MS-275 treatment of PA-induced C2C12 myotubes prevented insulin resistance and attenuated inflammatory signaling and cytokine production by restoring mitochondrial biogenesis and lipid oxidation. These effects were shown to be mediated by the inhibition of HDAC3 rather than HDAC1 and HDAC2. Similar results were seen *in vivo*, with MS-275 treatment of HF/HFr-fed mice ameliorating insulin resistance and reducing the expression of stress markers and TNF-α in skeletal muscle. Our study showed that HDAC inhibition is a promising therapeutic target for various metabolic diseases related to insulin resistance.

## Contribution to the Field Statement

Recently, epigenetic dysregulation has been proposed as a key contributor to the development of obesity and diabetes, with regulation of these events being a potential treatment target for these conditions. Previous studies have demonstrated the efficacy of histone deacetylase (HDAC) inhibitors for the treatment of various metabolic diseases, such as obesity, type 1 and type 2 diabetes mellitus, non-alcoholic fatty liver disease, and even chronic kidney disease. However, the effects of HDAC3 inhibition on free fatty acid-induced insulin resistance and inflammation in the C2C12 myotubes and skeletal muscle of high fat (HF)/high fructose (HFr) diet mice is not known. We found that MS-275 treatment of differentiated C2C12 myotubes improved PA-induced insulin resistance and decreased stress signals and inflammatory cytokine expression via the inhibition of JNK and NK-κB. Enhanced fatty acid oxidation and mitochondrial function were also observed following attenuation of lipotoxicity by MS-275. Similar beneficial effects were also seen following HDAC3 knockdown. In addition, *in vivo* treatment of HF/HFr-fed mice with MS-275 ameliorated insulin resistance, stress signals, and tumor necrosis factor-α expression in gastrocnemius muscle. Together, these results showed that MS-275 induced HDAC3 inhibition in skeletal muscle and may represent a promising candidate treatment for obesity and diabetes-related insulin resistance.

## Data Availability Statement

All data analyzed in this work will be made available by corresponding authors at the request of qualified researchers.

## Ethics Statement

All experiments were performed in accordance with the Ajou University Safety and Ethics guidelines. In particular, animal experiments were carried out according to the animal experiment procedure approved by the Animal Ethics Committee of Ajou University.

## Author Contributions

SJL, SEC, JYJ, and KWL contributed to the conception and design of the study. HBL, MWS, YK, HJK, THK, and JYJ were involved in data acquisition. YK, JYJ, and KWL contributed to data interpretation. All authors approved the paper for submission.

## Funding

This work was supported by Grant Nos NRF-2019R1A2C1003489 and NRF-2016R1D1A1B03930214 to KW Lee, NRF-2020M3A9E8024904 and NRF-2020R1I1A1A01075337 to JY Jeon from the National Research Foundation of Korea.

## Conflict of Interest

The authors declare that the research was conducted in the absence of any commercial or financial relationships that could be construed as a potential conflict of interest.

## Glossary

HDAC3: Histone deacetylase 3
HDAC2: Histone deacetylase 2
HDAC1: Histone deacetylase 1
PA: Palmitate
TG: triglyceride
ER: Endoplasmic reticulum
p-JNK: Phospho-C-JUN-N-terminal kinase
P-NK-κB: Phospho-nuclear factor-kappaB
DIO: Diet induced obesity
CHOP: C/EBP homologous protein
TFAM: mitochondrial transcription factor A
HF: High fat
HFr: High fructose
2-NBDG: 2-(N-(7-Nitrobenz-2-oxa-1,3-diazol-4-yl)Amino)-2-Deoxyglucos
TNF-α: Tumor necrosis factor-α
IL-1β: Interleukin-1β
IL-6: Interleukin-6
OCR: oxygen consumption rate
PGC1α: peroxisome proliferator activator receptor γ-coactivator 1α
PPARα: peroxisome proliferator-activated receptor alpha
MCAD: Medium-chain acyl-coenzyme A dehydrogenase
EHHADH: Enoyl-CoA Hydratase And 3-Hydroxyacyl CoA Dehydrogenase
SREBP1: Sterol regulatory element-binding protein 1
DGAT: Diglyceride acyltransferase
p-AKT: Phospho-protein kinase B
p-GSK3β: Phospho-glycogen synthase kinase 3 β
