# Supplementary material for: A Class I Histone Deacetylase Inhibitor Attenuates Insulin Resistance and Inflammation in Palmitate-Treated C2C12 Myotubes and Muscle of HF/HFr Diet Mice
Source: Front Pharmacol. 2020 Dec 10;11:601448. doi: 10.3389/fphar.2020.601448 (PMC7758468; doi:10.3389/fphar.2020.601448)
Supplement: Supplementary file 1 [file datasheet1.docx]

| TNFα | F | TCC AGG CGG TGC CTA TGT CT |
| --- | --- | --- |
|  | B | AAA TCG GCT GAC GGT GTG GG |
| IL1β | F | TCT CGC AGC AGC ACA TCA ACA |
|  | B | CCT GGA AGG TCC ACG GGA AA |
| IL6 | F | ATG CTG GTG ACA ACC ACG GC |
|  | B | AGG AGA GCA TTG GAA ATT GG |
| PPARα | F | CGA GGT GAA AGA TTC GGA AA |
|  | B | GGC CTT GAC CTT GTT CAT GT |
| PPARβ/δ | F | AAT GCG CTG GAG CTC GAT GAC |
|  | B | ACT GGC TGT CAG GGT GGT TG |
| MCAD | F | GAT CGC AAT GGG TGC TTT TGA |
|  | B | AGT TGA TTG GCA ATG TCT CCA |
| EHHADH | F | AAG CCG GGA GCC TTT CTG TG |
|  | B | CAT GAT GTG GGC TGG GGA GA |
| SREBP1 | F | GCT GTT GGC ATC CTG CTA TC |
|  | B | ATG CTG GAA GTG ACG GTG GT |
| DGAT | F | TGG TGT GTG GTG ATG CTG ATC |
|  | B | GCC AGG CGC TTC TCA A |
| SCD | F | CTC CAG TTC TTA CAC GAC CA |
|  | B | AGC CAC GGC GGG AAT TGT GA |
| PGC1α | F | GTC ATT CGG GAG CTG GAT GG |
|  | B | CAA CCA GAG CAG CAC ACT CT |
| TFAM | F | GCA CTT GAA ATG TGG GGA GT |
|  | B | CCT AAC CTA CAC CCC TGC AA |
| HDAC1 | F | TGC TGT GAA CTA CCC ACT GC |
|  | B | CAC TGC ACT AGG CTG GAA CA |
| HDAC2 | F | TGC TGT CAA TTT TCC CAT GA |
|  | B | GCG CTA GGC TGG TAC ATC TC |
| HDAC3 | F | TGC TTC AAT CTC AGC ATT CG |
|  | B | GTA GCC ACC ACC TCC CAG TA |

**Supplementary Table 1. List of primer sequence used for RT-PCR analysis in this study**

**Supplementary Figure**


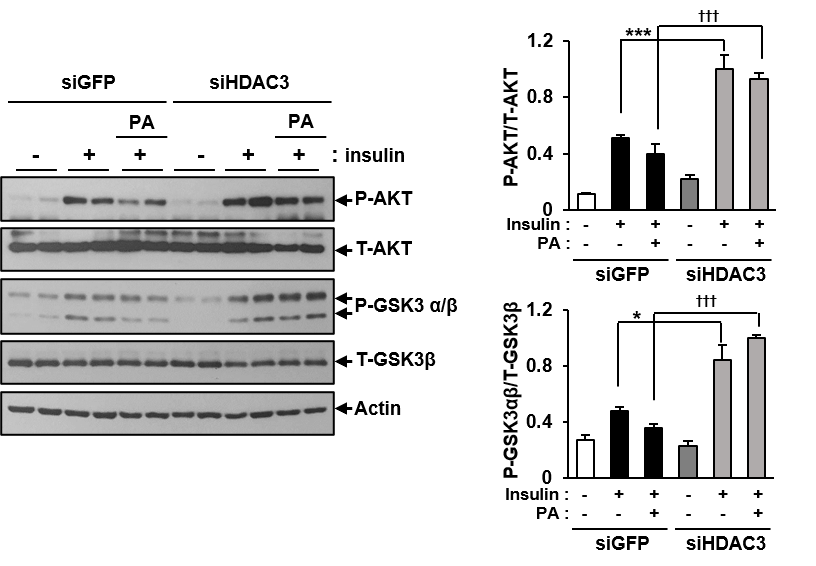


**Supplementary Figure 1.** MS-275 restored PA-reduced insulin signaling in C2C12 myotubes. HDAC3 siRNA transfected C2C12 myotubes were treated with 300 μM PA for 12 h and then 700 stimulated with 100 nM insulin for 20 min. Insulin signaling was determined by measuring the levels of p-AKT and p-GSK3β by Western blot. *p < 0.05; ***p < 0.001 vs. insulin-stimulated and GFP transfected cells, †††p < 0.001 vs. insulin-stimulated, GFP-transfected, and PA-treated cells.


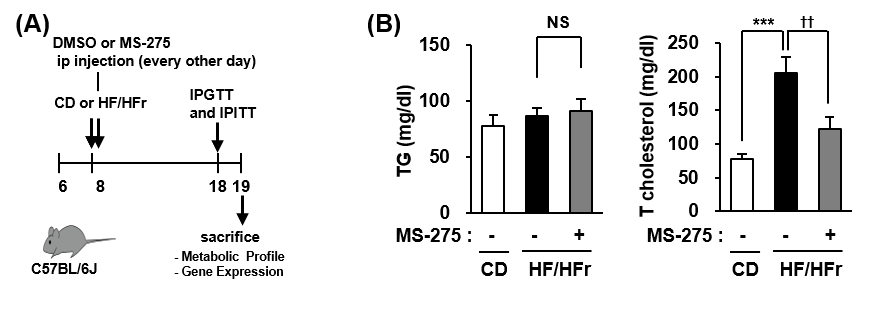


**Supplementary Figure 2.** (A) Animal experiment overview. DMSO or MS-275 was injected intraperitoneally into C57BL/6J mice fed a CD or HF/HFr diet every other day for 11 weeks. (B) To assess serum triglyceride (TG) and total cholesterol levels in C57BL/6J mice, Blood obtained from mouse heart was immediately centrifuged at 1,500xg for 15 min. Supernatant was collected and stored at -80 °C. Serum levels of TG and cholesterol were measured using triglyceride assay kit and total cholesterol assay kit (Roche Diagnostic International, Mannheim, Germany), according to the manufacturer’s instructions.
